# Complementary Roles of Cardiac Computed Tomography Angiography and Catheter Angiography Before Fontan Completion in Patients with Single-Ventricle Physiology: A Single-Center Experience

**DOI:** 10.3390/jcm15135286

**Published:** 2026-07-07

**Authors:** Burcu Çevlik, Ahmet Saki Oğuz, Demet Kangel, Kahraman Yakut, Muhammet Hamza Halil Toprak, İbrahim Cansaran Tanıdır, Serap Baş, Ali Can Hatemi, Erkut Öztürk

**Affiliations:** 1 Department of Pediatric Cardiology, Saglik Bilimleri UniversitBasaksehir Cam and Sakura City Hospital, Istanbul 34480, Turkey; ahmetsaki@gmail.com (A.S.O.); demetkangel@gmail.com (D.K.); kahramanyakut@gmail.com (K.Y.); muhammedhamzatoprak@hotmail.com (M.H.H.T.);; 2 Department of Radiology, Saglik Bilimleri University Basaksehir Cam and Sakura City Hospital, Istanbul 34480, Turkey; 3 Department of Pediatric Cardiovascular Surgery, Saglik Bilimleri University Basaksehir Cam and Sakura Hospital, Istanbul 34480, Turkey

**Keywords:** Fontan procedure, multimodality imaging, computed tomography angiography, catheter angiography

## Abstract

**Background**: In congenital heart disease patients with single-ventricle physiology, accurate anatomical and hemodynamic evaluation prior to the Fontan completion procedure is critical for surgical planning and long-term outcomes. Although conventional catheter angiography (CCA) remains the reference standard for hemodynamic evaluation, cardiac computed tomography angiography (CTA) has emerged as an increasingly valuable tool for anatomical assessment in the preoperative evaluation of Fontan candidates. **Objective**: To compare the anatomical and hemodynamic information provided by CTA and CCA before Fontan completion and to evaluate their complementary roles in the preoperative assessment of pediatric patients with single-ventricle physiology. **Methods**: This single-center, retrospective study included 32 patients with single-ventricular physiology who underwent staged palliation and Fontan completion. All patients underwent TTE and CCA. Twenty-three patients also underwent cardiac CTA with three-dimensional reconstruction. Anatomical and hemodynamic findings obtained from CTA and CCA were compared, with particular focus on extracardiac anatomy, collateral vessels, and findings relevant to surgical planning. **Results**: Cardiac CTA showed concordant detection of clinically significant anatomical findings identified by CCA, while providing a more comprehensive assessment of extracardiac anatomy. While CCA remained superior for hemodynamic assessment, CTA provided more comprehensive evaluation of extracardiac anatomy. In this cohort, catheterization findings did not alter the planned surgical strategy (0%, 95% CI 0–10.9%). **Conclusions**: CTA may provide valuable anatomical information for preoperative assessment and surgical planning; however, larger prospective multicenter studies are required before less invasive pre-Fontan evaluation strategies can be considered.

## 1. Introduction

The most severe part of the congenital heart disease spectrum consists of patients with univentricular physiology who do not have the appropriate physiology to maintain biventricular circulation [1]. The main aim of surgical palliation in this population is to maximize systemic blood oxygenation and create a physiology suitable for Fontan circulation by preserving the functional capacity of the systemic ventricle. The Fontan procedure refers to the final surgical stage in which systemic venous blood flow is redirected to the pulmonary circulation [2]. Patients with functionally single-ventricle physiology undergoing Fontan completion require detailed preoperative hemodynamic and anatomical evaluation using advanced imaging modalities such as CCA, cardiac magnetic resonance imaging, and cardiac CTA.

Fontan surgery is the final surgical step for most patients with a functionally single-ventricle and causes a significant change in cardiovascular physiology [2]. Transthoracic echocardiography remains the first-line imaging modality for the evaluation of these patients. Therefore, patients undergoing phased palliation often require advanced imaging techniques in the preoperative period. Traditionally, CCA has been considered the gold standard for both detailed anatomical and hemodynamic evaluation. However, the necessity of routine invasive hemodynamic evaluation in selected patients has been debated in recent years [3,4,5].

Although cardiac CTA is increasingly used in pre-Fontan assessment, the optimal integration of CTA and CCA in clinical practice is still not clearly defined. Studies directly comparing the anatomical and hemodynamic contributions of both methods in the same patient group before Fontan completion are limited. Therefore, the aim of this study is to comparatively evaluate the complementary roles of cardiac CTA and CCA in the preoperative assessment before the Fontan completion procedure in a pediatric patient group with single-ventricle physiology.

## 2. Materials and Methods

This study was planned as a single-center, retrospective study and conducted in accordance with the Declaration of Helsinki. The study was approved by the Ethics Committee of Istanbul Çam and Sakura City Hospital, Health Sciences University of Turkey (Protocol Code: 2025/307 and Date: 15 October 2025).

Between January 2022 and December 2025, 180 cases diagnosed with single-ventricle and scheduled for staged palliation were screened at our clinic. Children under 18 years of age with single-ventricle congenital heart disease who underwent Fontan completion surgery were included in the study. One hundred forty-eight patients were excluded due to being over 18 years of age, having not undergone third-stage palliation, and having undergone biventricular repair surgery.

All patients were evaluated with TTE during diagnosis and follow-up. All patients underwent conventional catheter angiography (CCA) before Fontan completion according to our institutional protocol. Cardiac CTA was not routinely performed throughout the entire study period. During the earlier years of the study period, CTA was used selectively, whereas its utilization gradually increased with expanding institutional experience and integration of advanced cardiac imaging into routine practice. As a result, 23 patients (71.9%) underwent both CTA and CCA and constituted the primary subgroup for comparative imaging analyses. The remaining patients underwent CCA without CTA and were included only in the overall cohort description. Clinically significant findings were defined as anatomical abnormalities with potential implications for Fontan candidacy, surgical planning, pulmonary blood flow, oxygen saturation, or the need for surgical or catheter-based intervention. These included Glenn pathway stenosis, significant collateral vessels, pulmonary artery abnormalities requiring reconstruction, and major vascular malformations. Small collateral vessels or minor vascular abnormalities not expected to alter management were classified as clinically insignificant findings.

All patients underwent routine transthoracic echocardiographic evaluation during diagnosis and follow-up. Echocardiographic evaluation was performed using a Philips EPIQ CVx Cardiac Ultrasound (Best, The Netherlands). Echocardiography was used for the assessment of cardiac anatomy, ventricular function, and valvular abnormalities and served as part of the standard preoperative evaluation. However, echocardiographic findings were not included in the primary comparative analyses between CTA and CCA.

Cardiac CTA was performed using a 640-slice scanner (Aquilion ONE, GENESIS Edition, Canon Medical Systems, Otawara, Japan) with prospective electrocardiographic triggering. All images were acquired in volumetric axial mode (rotation time: 0.35 s, scan range: 80–120 mm). Low tube voltage (80 kV) and automatic exposure control were used to reduce radiation dose. All patients received 1.5 mL of an iodinated contrast agent (Kopaq 350 mg/mL, Onko&Kocsel Pharmaceuticals, Kocaeli, Turkey) per kg, followed by a 10–20 mL saline infusion. The injection rate was set at 0.7–0.9 mL/kg. All images were acquired during the first contrast pass through the anatomical structures of interest. Targets for the center of the acquisition window were defined as 45% of the R-R interval in patients (all patients with a heart rate > 90 bpm). Images were reconstructed with a slice thickness of 0.5 mm and evaluated using multiplane reconstruction, maximum intensity projection, and three-dimensional volume rendering techniques.

Cardiac catheterization procedures were performed under general anesthesia using a dual-wing angiography system (Philips^®^ AZURION 7 B12, Best, The Netherlands). The femoral artery, femoral vein, and jugular vein were used as vascular access sites. Hemodynamic data, including intracardiac pressures and oxygen saturation levels, were recorded from multiple heart chambers, the aorta, and the vena cavae using fluid-filled catheters. During the angiographic evaluation, an intravenous iodinated contrast agent (Kopaq 350 mg/mL) was used at a dose not exceeding 4 mL/kg. Mean pulmonary artery pressure, pulmonary vascular resistance/systemic vascular resistance (PVR/SVR) ratio, Qp/Qs ratio, and MacGoon and Nakata indices were calculated.

Cardiac CTA examinations were interpreted in routine clinical practice by two pediatric cardiovascular radiologists with 5 and 11 years of experience, respectively. Conventional catheter angiography studies were interpreted by experienced pediatric cardiologists at the time of catheterization. Data used in the present study were obtained retrospectively from clinical imaging reports and medical records. No formal blinded re-evaluation of CTA or CCA images was performed for study purposes.

### Statistical Analysis

Descriptive and inferential statistical methods were used for data analysis. The distribution of continuous variables was evaluated using Kolmogorov–Smirnov and Shapiro–Wilk tests. Variables showing a normal distribution were analyzed using parametric tests, while variables not showing a normal distribution were analyzed using non-parametric tests. Categorical variables were presented as numbers and percentages. Anthropometric measurements were analyzed using percentile values and standard deviation scores (z-score). Relationships between the two imaging modalities were evaluated using the Chi-square test. Agreement between CTA and CCA for categorical imaging findings was assessed using Cohen’s kappa coefficient. Kappa values were interpreted as follows: <0.20 poor, 0.21–0.40 fair, 0.41–0.60 moderate, 0.61–0.80 good, and >0.80 excellent agreement. Sensitivity and specificity of CTA were calculated using CCA as the reference standard in patients who underwent both imaging modalities (n = 23). Due to the exploratory nature of the study and the limited sample size, agreement analyses were interpreted descriptively. All statistical analyses were performed using SPSS version 15.0 (SPSS Inc., Chicago, IL, USA), and a *p* < 0.05 was considered statistically significant.

## 3. Results

**Figure 1:**
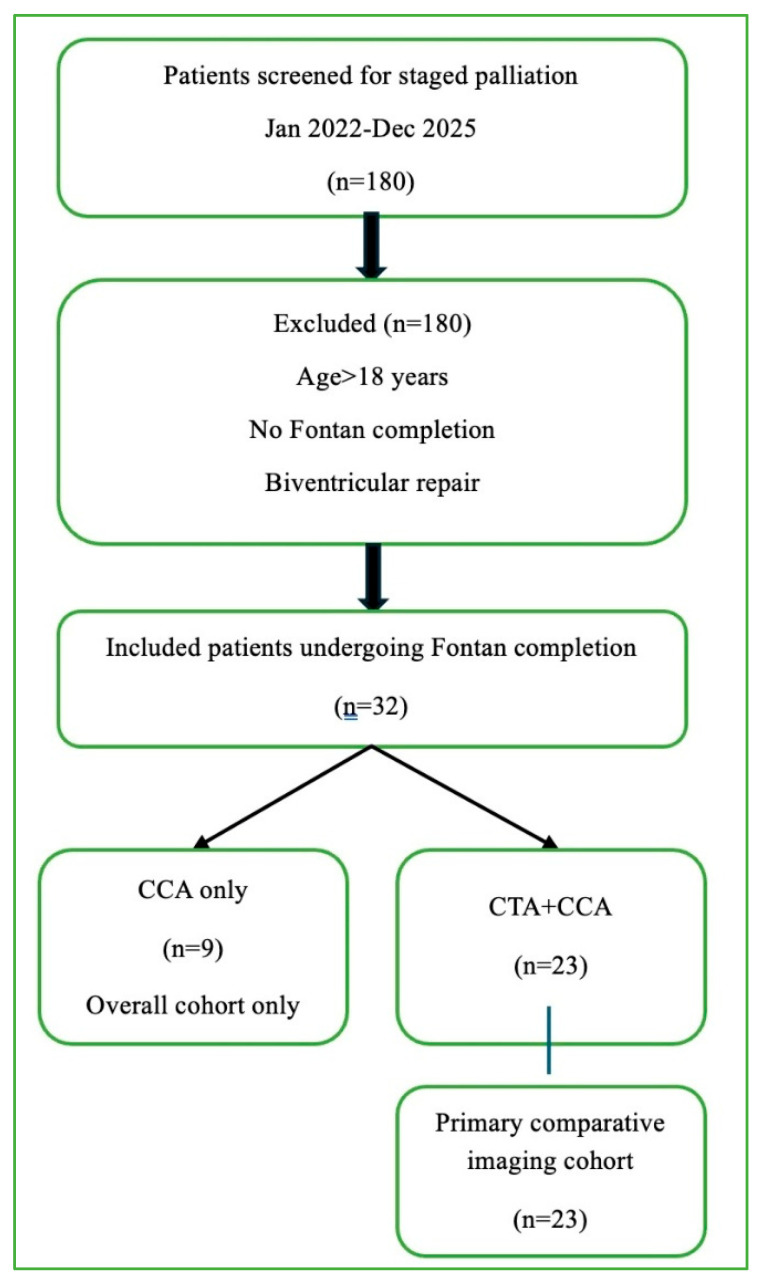
Study flow diagram showing patient screening, exclusion, inclusion, and allocation to imaging modalities.

A total of 32 patients were included in the study (Figure 1), 56.3% (n = 18) of whom were male. The median age of the patients was 52 months. The median body weight was 14 kg, the median height was 96 cm, and the median body mass index was 14.9 kg/m^2^. The median age at the time of Fontan surgery was 41.5 months. Among the 32 patients included in the overall Fontan cohort, 23 (71.9%) underwent both cardiac CTA and CCA and constituted the primary imaging comparison subgroup. Comparative analyses of anatomical findings and agreement between imaging modalities were performed exclusively within this subgroup.

In the second stage of palliation, 81.2% (n = 26) of the cases underwent unilateral bidirectional Glenn surgery, and in the third stage of palliation, 37.5% (n = 12) of the cases underwent extracardiac Fontan surgery. The demographic characteristics of the cases are summarized in Table 1.

The most frequent diagnoses were TA-VA concordance (21.9%, n = 7), double-inlet left ventricle–VA discordance (DILV-VA discordance) (12.5%, n = 4), and transposition of the great arteries–ventricular septal defect–pulmonary stenosis (TGA-VSD-PS) (12.5%, n = 4). There was no statistically significant difference between the diagnoses and the types of Glenn and Fontan operations performed (*p* > 0.05). All patients underwent CCA prior to third-stage palliation. Overall, 71.9% (n = 23) of patients were additionally evaluated with cardiac CTA.

**Figure 2:**
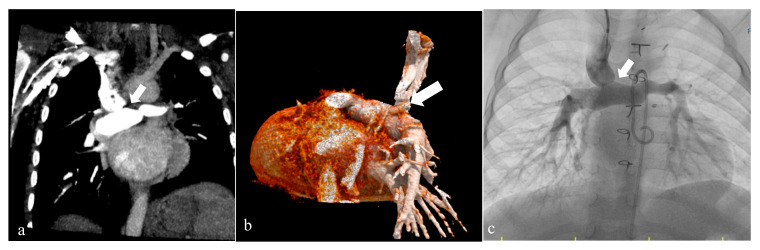
Four-year-old patient with IVS-PA and stenosis in the Glenn shunt. (**a**,**b**) Visualization of the stenosis in the Glenn shunt with CTA and 3D-CTA. (**c**) Visualization of the stenosis in the Glenn shunt with catheterization.

**Figure 3:**
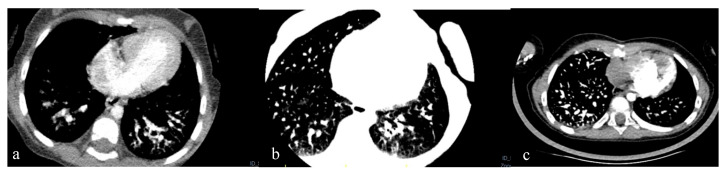
Images of lung parenchymal abnormalities in cases using CTA. (**a**) Centric nodules and ground-glass opacity in the lung parenchyma of a patient diagnosed with TA-VA concordance. (**b**) Sequelae lesions and emphysematous changes in the lung parenchyma of a patient diagnosed with DILV-VA discordance. (**c**) Air trapping in the lung parenchyma of a patient diagnosed with DILV-DORV.

**Figure 4:**
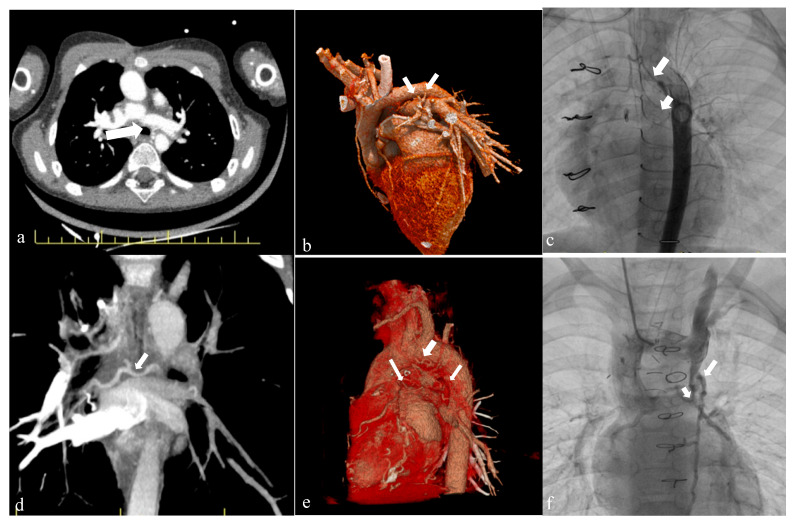
Demonstration of multiple collaterals (**a**–**c**): In a patient diagnosed with TA-VA discordance, collaterals originating from the descending aorta and arch and extending to both hila are shown with CTA and CCA. (**d**–**f**): In a patient diagnosed with VSD-PA, collaterals originating from the left innominate artery and arch are shown with CTA and CCA.

The median dose-length product of cardiac CTA procedures was 45.2 mGycm (35.4–462.8 mGycm), and the median corrected radiation dose was 0.89 mSv (0.61–4.59 mSv). In one case, stenosis was observed in both the CCA and CTA at the Glenn anastomosis (Figure 2). When the lung parenchyma was evaluated with cardiac CTA after recurrent surgery, no pathology was observed in twenty cases. One case showed air trapping, another showed ground-glass opacity and centrilobular nodules, and a third showed emphysematous changes (Figure 3). Cardiac CTA revealed a single venovenous (VV) collateral in 12.5% (n = 4) and multiple venovenous collaterals in 12.5% (n = 4) of the cases (Figure 4). One patient had an arteriovenous malformation (AVM), and two cases had coronary cameral fistulas.

In patients evaluated with CCA, 50% (n = 16) had VV collaterals (28.1% (n = 9) two or more and 21.8% (n = 7) single collaterals) (Figure 4), 28.1% had aortopulmonary collateral arteries (APCA), 12.5% had arteriovenous (AV) fistulas, and 9.3% had VV fistulas. Fistulas and collaterals most frequently originated from the left innominate vein and descending aorta (Figure 4). No statistically significant relationship was found between diagnosis and collateral development (*p* = 0.289).

The median pulmonary artery pressure measured by conventional catheter angiography was 14 mmHg, the median pulmonary/systemic vascular resistance (PVR/SVR) ratio was 0.1, the median Qp/Qs ratio was 0.67, the median MacGoon index was 2.03, and the median Nakata index was 245.5 mm^2^/m^2^. Following angiographic evaluation, it was found that 15.6% (n = 5) of the cases required additional pulmonary artery reconstruction, which was also confirmed by cardiac CTA. No statistically significant difference was found between hemodynamic measurements and the type of Fontan operation performed (*p* > 0.05). Importantly, no patient underwent a change in planned Fontan strategy based solely on catheterization findings. The rate of surgical method change solely due to catheterization was calculated as 0% (95% confidence interval: 0–10.9).

All patients underwent standard CCA, while a subset of patients also underwent CTA. Comparative analyses were performed within this subgroup (n = 23) that received both imaging methods (Table 2). In our cohort, CTA identified all clinically significant anatomical findings that were also detected by CCA (κ =1.00, *p* < 0.001). Nevertheless, the number of clinically significant abnormalities was small, limiting the robustness of agreement estimates and precluding definitive conclusions regarding diagnostic performance. These findings suggest that cardiac CTA can provide sufficient anatomical preoperative information in selected patients. However, the number of such findings was limited, and therefore these results should be interpreted cautiously. However, agreement was weak for hemodynamically insignificant findings (κ = −0.017), indicating limited agreement between the two modalities in detecting small lesions, with CCA showing higher sensitivity.

Postoperative outcomes were recorded descriptively. Minor postoperative complications occurred in 13 patients, including pleural effusion, wound-related complications, and rhythm disturbances. Three patients died during follow-up. Owing to the limited sample size, no significant associations were identified between imaging findings and postoperative outcomes.

## 4. Discussion

Fontan surgery represents the final surgical step for patients with a functionally single ventricle and leads to a radical change in cardiovascular physiology. The main objective of this study is to demonstrate the clinical value of a multimodal imaging approach before third-stage palliation in pediatric patients with single-ventricle physiology and evaluate the complementary roles of CTA and CCA in contemporary pre-Fontan assessment. In the literature, the most frequent diagnoses in patients undergoing single-ventricle palliation are reported to be TA, DILV and DORV [4,6]. Similarly, in our study, the most frequent diagnoses were TA and DILV, and our patient profile was found to be consistent with the literature.

Detailed anatomical evaluation after second-stage palliation is critical for planning Fontan completion surgery. Transthoracic echocardiography remains the first-line method for evaluating Fontan circuit patency, single ventricular function, and valve pathologies [7]. However, poor acoustic windows and complex anatomies can make it difficult to obtain sufficient information in some cases. Therefore, in recent years, there has been a significant increase in the use of transesophageal echocardiography, cardiac magnetic resonance imaging, and cardiac CTA [8]. Traditionally, conventional catheter angiography has been considered the gold standard in the evaluation of these anatomical structures. However, advances in imaging technology have led to an increasing use of non-invasive techniques offering lower radiation dose, less contrast requirement, and shorter procedure time [9]. In the present study, the median dose-length product in patients evaluated with CTA was 45.2 mGycm (35.4–462.8 mGycm) and the median corrected radiation dose was 0.89 mSv (0.61–4.59 mSv), indicating exposure to very low doses of radiation.

As suggested by Prakash et al. [10], using TTE and non-invasive advanced imaging methods first in selected patients and only using CCA in cases where high risk is predicted is considered a rational approach. In our study, CCA was performed on all patients for hemodynamic evaluation, and cardiac CTA was used in addition to the CCA in 71.9% of the patients. The fact that CCA was performed on all patients before Fontan due to the need for hemodynamic evaluation in our center indicates that invasive evaluation still maintains its importance.

Cardiac CTA has emerged as a powerful alternative, especially in anatomical assessment, thanks to its high spatial resolution, fast imaging time, and non-invasive nature [11,12,13]. Cardiac CTA provided comprehensive visualization of extracardiac anatomy, including pulmonary arteries, Glenn pathways, collateral vessels, and lung parenchyma. In addition to identifying clinically relevant vascular abnormalities such as Glenn stenosis, CTA allowed three-dimensional assessment of complex postoperative anatomy, which may facilitate surgical planning. These findings are consistent with previous reports highlighting the value of CTA as a rapid and high-resolution imaging modality in patients with single-ventricle physiology. Furthermore, pre-procedural assessment with cardiac CTA could facilitate procedural planning during catheterization by providing insight into detailed anatomy. We observed that the use of cardiac CTA has been increasing in our center over time and is becoming more integrated into clinical practice.

One of the important factors affecting long-term prognosis in patients with single-ventricle physiology is collateral development. In the literature, the most common types of collaterals are reported to be APCAs and VV collaterals [14]. Cardiac CTA identified all clinically significant findings detected by CCA in this cohort (K = 1.00, *p* < 0.001); however, the number of such findings was limited. However, agreement was lower for small collateral vessels and fistulous communications, reflecting the higher sensitivity of catheter angiography for detecting subtle lesions. The negative kappa value observed for fistula detection should be interpreted cautiously because of the very small number of events and the resulting instability of agreement statistics. These findings suggest that CTA may reliably identify clinically relevant anatomical abnormalities, while CCA retains an important role in the evaluation of minor vascular abnormalities and invasive hemodynamic measurements. In addition to identifying findings that directly influence surgical planning, CTA may also detect subtle extracardiac anatomical abnormalities, such as small collateral vessels or anomalous venous structures, which may not alter the immediate surgical strategy but remain valuable for longitudinal follow-up and future clinical management.

Despite the increasing role of advanced non-invasive imaging, direct assessment of pulmonary artery pressure, transpulmonary gradients, and pulmonary vascular resistance remains beyond the capability of CTA. Although CTA provides detailed anatomical information, it cannot directly assess key hemodynamic parameters required for Fontan decision-making. Successful Fontan circulation depends not only on favorable anatomy but also on preserved ventricular function, competent atrioventricular valves, low pulmonary vascular resistance, and acceptable pulmonary artery pressures. Therefore, conventional catheterization remains indispensable in selected patients requiring comprehensive hemodynamic assessment before Fontan completion. Consequently, invasive catheterization continues to be an essential component of pre-Fontan assessment in many patients, particularly those with suspected elevated pulmonary vascular resistance, ventricular dysfunction, cyanosis, or complex anatomy. Therefore, our findings should not be interpreted as evidence that CTA can replace catheterization, but rather that it may complement and potentially streamline preoperative evaluation in carefully selected patients.

Although survival has significantly increased after the Fontan procedure, complications such as pleural effusion, arrhythmias, thrombosis, protein-losing enteropathy, and Fontan insufficiency are still significant causes of morbidity and mortality [15,16,17]. Pleural effusion is one of the most studied of these complications in the literature and has been shown to be more frequent in those who undergo Fontan surgery without fenestration [18]. The most frequent complication observed in our study was pleural effusion, a finding consistent with the increased risk of pleural effusion after a non-fenestration Fontan procedure reported in the literature (*p* = 0.038).

An interesting finding of our study was that catheterization findings did not alter the planned surgical strategy in any patient. Although this observation should be interpreted cautiously given the limited sample size and retrospective design, it supports ongoing discussions regarding the selective rather than universal use of invasive assessment before Fontan completion. These approaches provide preoperative surgeons with the ability to examine the cardiac anatomy in detail and minimize potential intraoperative difficulties by evaluating the pulmonary arteries, bidirectional Glenn pathway and collaterals [19]. This observation may reflect patient selection, institutional practice patterns, or the increasing role of advanced non-invasive imaging. Furthermore, successful Fontan circulation depends on multiple factors including ventricular function, atrioventricular valve competence, pulmonary artery development, pulmonary vascular resistance, and patient-specific anatomical characteristics. In addition, factors such as extracardiac conduit selection and conduit size may influence Fontan hemodynamics and long-term outcomes and therefore remain important considerations during preoperative surgical planning. These parameters remain critical determinants of Fontan candidacy and long-term outcomes and should be considered alongside imaging findings during preoperative assessment.

Our findings support a complementary rather than competitive relationship between CTA and CCA in pre-Fontan assessment. This concept is consistent with contemporary multimodality imaging strategies in congenital heart disease, which emphasize integration of complementary imaging modalities rather than reliance on a single technique [20]. CTA provides detailed visualization of extracardiac anatomy, collateral vessels, and postoperative vascular pathways, whereas CCA remains indispensable for invasive hemodynamic assessment in selected patients [21]. In addition to findings directly influencing surgical planning, CTA may identify subtle extracardiac anatomical abnormalities that are relevant for longitudinal follow-up and future clinical management. In our cohort, the mean effective radiation dose of CTA was low. Together with advances in pediatric CT technology, this relatively low radiation exposure may represent an important advantage for children with congenital heart disease who frequently undergo repeated imaging throughout long-term follow-up. Accordingly, integration of both modalities may facilitate comprehensive preoperative evaluation and surgical planning.

## 5. Study Limitations

This study has several limitations. First, it was a retrospective single-center study with a relatively small sample size, which limits the generalizability of the findings. Second, cardiac CTA was not performed in all patients, as its use evolved over the study period, introducing the potential for selection bias. Third, the number of clinically significant findings was low, limiting the robustness of agreement statistics and diagnostic performance estimates. Fourth, imaging data were derived from routine clinical reports, and no formal blinded re-evaluation of CTA or CCA studies was performed. Consequently, observer bias cannot be excluded, as image interpretation may have been influenced by prior clinical information and routine clinical practice. This limitation may have affected the observed agreement between CTA and CCA and should be considered when interpreting our findings. Finally, quantitative CTA-derived anatomical measurements were not systematically available and therefore could not be analyzed. Comparative procedural parameters such as CCA radiation exposure, fluoroscopy time, contrast burden, and CTA sedation requirements were not consistently available and therefore could not be analyzed. Accordingly, the findings should be interpreted as exploratory and hypothesis-generating, requiring confirmation in larger prospective multicenter studies.

## 6. Conclusions

In conclusion, our findings support the use of a multimodality imaging strategy before Fontan completion in patients with single-ventricle physiology. Cardiac CTA provides detailed anatomical assessment of extracardiac structures, pulmonary arteries, and collateral vessels, whereas CCA remains indispensable for hemodynamic evaluation. Rather than competing modalities, CTA and CCA should be considered complementary tools in preoperative decision-making. Future prospective multicenter studies are needed to determine whether selected patients may safely undergo a less invasive pre-Fontan assessment strategy.

## Figures and Tables

**Table 1:** Demographic characteristics of the cases.

Patient Characteristics	n	%	Median	Range
Sex				
Male	18	56.3		
Female	14	43.8		
Median Age (months)			52	35–165
Median body weight (kg)			14	10.4–50
Median height (cm)			96	80–161
Median body mass index			14.9	9–22
Diagnosis				
TA-VA concordance	7	21.9		
DILV-VA discordance	4	12.5		
TGA-VSD-PS	4	12.5		
IVS-PA	2	6.3		
VSD-PA	2	6.3		
Tricuspid hypoplasia	2	6.3		
TA-VA discordance	2	6.3		
cTGA	2	6.3		
HLHS	2	6.3		
DILV-DORV	1	3.1		
DIRV-DORV	1	3.1		
DORV-PA	1	3.1		
DILV-VA concordance	1	3.1		
DORV-cAVSD	1	3.1		
Glenn operation types				
Bidirectional	27	84.4		
Bilateral bidirectional	4	12.5		
Bilateral unidirectional	1	3.1		
Fontan operation types				
Extracardiac Fontan	12	37.5		
Fenestrated Fontan	11	34.4		
Classic Fontan	6	18.8		
Extracardiac fenestrated Fontan	3	9.4		
Death (+)	3	9.4		

Abbreviations: cAVSD, Complete atrioventricular septal defect; cTGA, Corrected TGA; DILV, Double-inlet left ventricle; DORV-PA, Double-outlet right ventricle–pulmonary atresia; HLHS, Hypoplastic left heart syndrome; IVS-PA, pulmonary atresia with intact ventricular septum; PS, pulmonary stenosis; VA, ventriculoarterial; VSD-PA, ventricular septal defect–pulmonary atresia; TA, tricuspid atresia.

**Table 2:** Comparison of CTA and CCA performed together in 23 patients.

		CTA (n = 23)	CCA (n = 23)	Kappa (κ) Value	*p* Value	Sensitivity	Specificity
Glenn shunt	Normal	95.6% (n = 22)	95.6% (n = 22)	K = 1.00	*p* < 0.001	100%	100%
	Stenosis	4.3% (n = 1)	4.3% (n = 1)				
Hemodynamically insignificant collateral	No	73.9% (n = 17)	65.2% (n = 15)	K = −0.017	*p* = 0.91	75%	100%
	Yes	24% (n = 6)	34.7% (n = 8)				
Hemodynamically significant collateral	No	91.3% (n = 21)	91.3% (n = 21)	K= 1.00	*p* < 0.001	100%	100%
	Yes	8.7% (n = 2)	8.7% (n = 2)				
Fistula	No	91.3% (n = 21)	78.2% (n = 18)	K = −0.53	*p* = 0.63	50%	100%
	Yes	8.7% (n = 2)	21.7% (n = 5)				

## Data Availability

The data presented in this study are available from the corresponding author upon reasonable request. The data are not publicly available because they contain information that could compromise patient privacy.
